# Creative thinking in the teaching of Chinese language and literature in colleges from the perspective of educational psychology

**DOI:** 10.3389/fpsyg.2022.1018289

**Published:** 2022-10-13

**Authors:** Li Zhong

**Affiliations:** College of Humanities and Social Sciences, Heilongjiang Bayi Agricultural University, Daqing, China

**Keywords:** educational psychology, Chinese language, literature major, professional education, creative application, organizational psychology

## Abstract

In the major of Chinese language and literature, the reading of Chinese language and literature is the most basic part. With the development of internet technology, the teaching of Chinese language and literature has also ushered in new changes. In order to better innovate the teaching methods of Chinese language and literature education according to the emerging internet technology, this study applies the scaffolding teaching model based on educational psychology to Chinese language teaching. Firstly, the educational psychology and scaffolding teaching theory are analyzed, and a scaffolding teaching model of educational psychology is proposed. Secondly, this model is applied to the teaching of Chinese language and literature majors in colleges, and teaching experiments are carried out. The results show that the Chinese language and literature reading anxiety of the students in the experimental class has been alleviated to a certain extent. After using the scaffolding teaching model based on human-computer interaction and educational psychology to conduct teaching experiments, the average reading anxiety of the experimental class is 67.13 points, and the average reading anxiety of the control class is about 76.52 points. The average post-test reading anxiety of the experimental class is 9.39 points lower than that of the control class. The students’ Chinese language and literature scores improve after the experiment. Therefore, the scaffolding teaching model based on educational psychology can be used in the teaching of Chinese language and literature majors in colleges and universities. This study can realize the effective teaching inside and outside the classroom of Chinese language and literature in colleges and universities, promote the deep integration of educational psychology and the teaching of Chinese language and literature, and provide a new teaching idea for Chinese language and literature teachers.

## Introduction

### Research background and motivations

China’s education system has become increasingly perfect. Chinese language and literature is a unique humanity in China, the curriculum contains a unique traditional Chinese culture (Chen, 2021). Under the background of more and more education talents, employers are more willing to choose compound talents when recruiting talents. The education of Chinese language and literature will face greater challenges. Therefore, it is necessary to carry out a certain degree of reform in the teaching of Chinese language and literature, combining theory with practice. In addition, the major in Chinese language and literature has a strong particularity, and it is necessary to correctly face the practical value of the major in Chinese language and literature. And reading is considered to be the most basic of the four basic skills because it is an important way and skill for students to acquire knowledge (Cho et al., 2021). In addition, reading is the main way to improve learners’ comprehensive language ability, but how to use limited reading resources to obtain useful information in a short time has become a difficult problem for students (Relyea and Amendum, 2020). The current situation of Chinese language and literature reading teaching in Chinese universities is unsatisfactory. For students, they are not very interested in Chinese language reading, and there is a certain degree of reading anxiety, lack of self-confidence, and lack of autonomy in the reading process (Erbeli and Joshi, 2022). For teachers, their teaching methods are mainly based on traditional teaching methods, ignoring students’ emotional factors in the reading process. In addition, teachers’ feedback cycle is relatively long, so students’ questions cannot be answered in time. Teachers have begun to use computers and the internet to assist Chinese language and literature teaching, but they lack initiative (Yudhana, 2021). The key to solving this problem is how to cultivate and improve students’ Chinese language and literature reading ability or reading strategies.

### Research objectives

Chinese language reading teaching is undoubtedly an important way for college students to improve their Chinese language reading ability and strategies. It is an urgent task to seek an effective way to strengthen Chinese language reading teaching (Hughes et al., 2019). Therefore, college Chinese language reading teaching urgently needs a new teaching mode. Scaffolding teaching has obviously become an important direction of current classroom teaching research. Scaffolding teaching mode takes learners as the classroom center and helps learners to better complete learning tasks. However, this mode does not pay enough attention to students’ extracurricular autonomous learning, which will gradually weaken students’ interest in reading (Huang, 2022).

Based on this, the internet and educational psychology are attempted to integrate into the scaffolding teaching mode, and a scaffolding teaching mode based on the internet and educational psychology is proposed. This model is applied to the teaching of Chinese language reading in colleges and universities to achieve effective teaching within and outside the classroom of Chinese language reading in colleges and universities and to promote the integration of creative application of internet technology and Chinese language and literature teaching to provide a new teaching idea for the majority of college Chinese language and literature teachers, guide learners to maintain an optimistic attitude to learn, reduce tension and anxiety, and fully improve their learning ability. The innovation lies in the analysis of Chinese language and literature education from the perspective of educational psychology. Meanwhile, the problem is refined into Chinese language reading teaching, which makes the research results more detailed and reliable.

The research framework is: The scaffolding teaching mode based on educational psychology is applied to the teaching of Chinese language and literature reading, aiming to improve students’ reading interest and improve their reading level. Firstly, the theory of educational psychology and scaffolding teaching is analyzed, and a scaffolding teaching mode of educational psychology is proposed. The teaching of this mode is divided into three processes, namely pre-class teaching, classroom teaching, and after-class teaching. Secondly, this model is applied to the teaching of Chinese language and literature majors in colleges. Students from two classes of Shaanxi Normal University in Xi’an are selected as the research objects, and a one-semester teaching experiment is conducted. The reading result data of language and literature are analyzed using SPSS24.0 software after the experiment.

## Literature review

In order to enable students to learn the Chinese language and literature better, researchers are constantly optimizing the teaching mode of Chinese language and literature majors. Gao R. (2021) combined constructivist theory with teaching and proposed a new teaching model based on qualitative analysis and modular construction. This new teaching mode can not only broaden teachers’ teaching ideas and improve teaching efficiency, but also help students explore the law of learning cognition, construct vocabulary systems, improve teaching efficiency, and effectively stimulate their potential in the short term (Gao R., 2021). Sun et al. (2021) believed that the influence of students’ learning perception can be explored by providing digital games. A total of 141 primary school students and 4 mathematics teachers participates in the experiment, and qualitative data are collected through classroom observation and student interviews (Sun et al., 2021). Kim (2021) explored the impact of scaffolding on the Chinese students’ learning autonomy and academic performance. The research results show that first, compared with traditional teaching, scaffolding teaching is effective in learning autonomy. Second, scaffolding also has a statistically significant effect on students’ academic performance (Kim, 2021). Most colleges and universities use computer network technology to improve management efficiency and teaching quality, deepen the teaching mode of Chinese language and literature, accelerate the development of college teaching mode, create a good campus environment, and improve the level of information management in running schools. The current society’s demand for talents is not only the mastery of knowledge but also innovative thinking and the ability to quickly solve problems using analysis. This requires teachers to improve their own quality and skills and innovate teaching methods (Gao Y., 2021).

In a word, there are many studies on Chinese language teaching mode, and the mode adopted is basically mixed teaching, but there are not many studies on the teaching mode of Chinese language reading. Reading ability is one of the important criteria for measuring Chinese language learning of college students. However, most students generally have anxiety when reading articles, which leads to poor reading effects and reduced learning efficiency (Qian and Lau, 2022). In order to alleviate or eliminate students’ reading anxiety, the scaffolding teaching model is applied based on the internet and educational psychology to Chinese language and literature reading teaching for experimental research to explore the effectiveness of the new teaching model in actual teaching activities. It is hoped that the research results will play a certain role in improving the current situation of Chinese language and literature reading teaching in colleges.

## Research methodology

### Scaffolding instruction analysis

**FIGURE 1:**
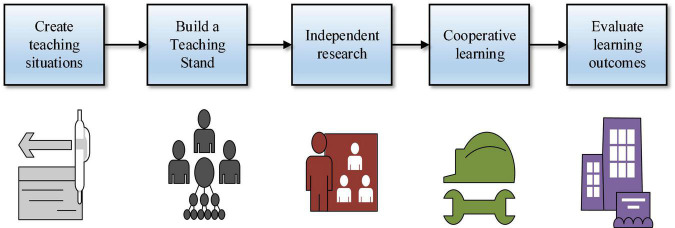
Scaffolding instruction steps.

The exact concept of scaffolding instruction is that teachers should provide students with a “scaffold” that is conducive to an effective understanding of knowledge when teaching, and use the “scaffold” to further enable students to understand the teaching content at a deeper level (Vo et al., 2022). Scaffolding instruction is not only a teaching idea but also a teaching mode. Scaffolding instruction is a teaching model that takes students as the main body, based on students’ knowledge level, and aims at cultivating students’ knowledge skills and innovation ability to further release and exert students’ potential (Onah, 2022). In this mode, teachers should pay special attention to building a teaching classroom culture of cooperation, communication, discussion, and competition so that students can maximize their initiative, enthusiasm, and creativity under the subtle influence of this culture, and ultimately greatly improve their overall quality (Widhiasih et al., 2022). The specific steps of scaffolding instruction are shown in Figure 1.

As shown in Figure 1, scaffolding instruction generally consists of related steps such as creating teaching scenarios, building teaching scaffolding, independent exploration and research, cooperative and mutual learning, and evaluating learning effects (Lee, 2018). The creation of a teaching situation means that teachers should set up a teaching theme that contains the main knowledge that students need to learn and all the main problems that students need to solve. The construction of teaching scaffolding refers to that teachers should take the knowledge content that students need to learn as the main development area, put forward relevant problems and understand some important knowledge concepts in textbooks as the basic point to build teaching scaffolding, and lay the foundation for students’ independent exploration and mutual learning discussion (Reynolds and Daniel, 2018). The evaluation of the learning effect is the most important step in scaffolding instruction. Teachers’ evaluation of students’ learning should be divided into two parts: one is the evaluation of the whole group, and the other is the evaluation of students’ comprehensive performance in the group (Liu et al., 2022).

### Teaching mode of Chinese language and literature based on scaffolding instruction

The practical research of scaffolding instruction mode in the classroom of Chinese language and literature major has achieved fruitful results, but there is little research on how to carry out scaffolding instruction activities outside the classroom (Chang et al., 2022). Teachers should extend the scope of teaching activities beyond the classroom through the Internet teaching platform with computer-aided support, optimize the scaffolding instruction activities, enrich the scaffolding instruction mode, and increase the timeliness and interactivity of teaching (Farida and Rozi, 2022). Therefore, combined with the existing scaffolding instruction mode and actual teaching situation based on the Internet and educational psychology, the innovative application of scaffolding instruction in the teaching of Chinese language and literature in universities. The scaffolded Chinese language and literature teaching process based on educational psychology is divided into three teaching processes: pre-class teaching, classroom teaching, and post-class teaching (Ivcevic et al., 2022).

#### Pre-class teaching

**FIGURE 2:**
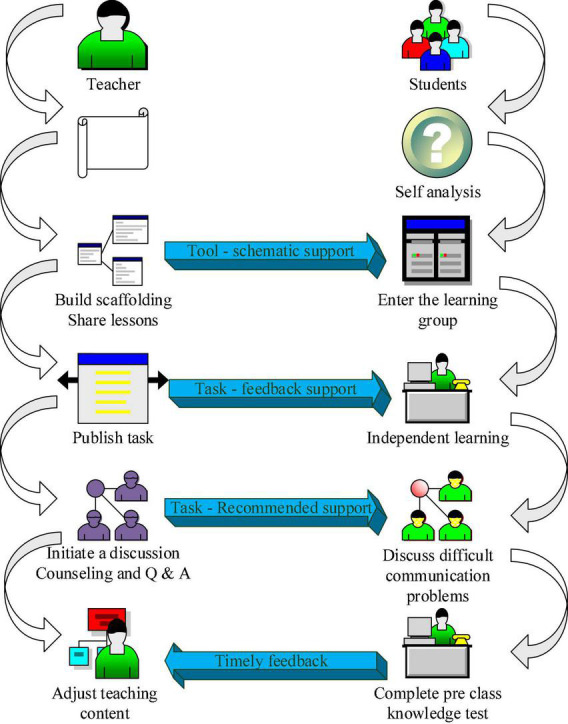
The pre-class teaching process.

The primary process of the scaffolding Chinese language and literature teaching model based on the Internet and educational psychology is pre-class teaching. During this process, students’ learning effect directly affects the effect of classroom teaching (Piamsai, 2020). Students should complete the meaningful construction of new knowledge before class, which will help them carry out in-depth learning in class, so the design of online teaching activities is very important (Mahan, 2022). The pre-class teaching process is shown in Figure 2.

Figure 2 shows that the preliminary analysis is the teacher’s teaching idea based on the scaffolding theory. Starting from the students’ zone of proximal development, the teaching content is decomposed into multiple learning tasks, so that in the teaching process, according to the specific learning situation of the students, the appropriate learning scaffold is constructed to achieve effective teaching, stimulate the enthusiasm of students’ learning, and improve students’ learning self-confidence. Building a scaffold and sharing courses means that teachers provide students with pre-class teaching resources, such as documents, pictures, courseware, and web links according to their learning tasks, create courses on the learning platform and upload teaching resources (Li and Tao, 2022). Publishing learning tasks means that teachers assign pre-class online learning tasks for students based on students’ original learning ability and actual teaching content. Initiating discussions and tutoring and answering questions means that student’s feedback on their knowledge points and questions for discussion in the learning exchange group. Teachers build a scaffold in the process and guide them in time (Lin et al., 2021). The pre-class knowledge test is that teachers design test questions according to the learning content and the actual situation of students, and students complete the pre-class knowledge test and submit them to the learning platform as required. Timely adjustment of teaching content is the design of teachers to adjust classroom teaching activities in time according to students’ completion of pre-class preview tasks and the results of pre-class knowledge tests (Demirkol, 2022).

#### Classroom teaching

**FIGURE 3:**
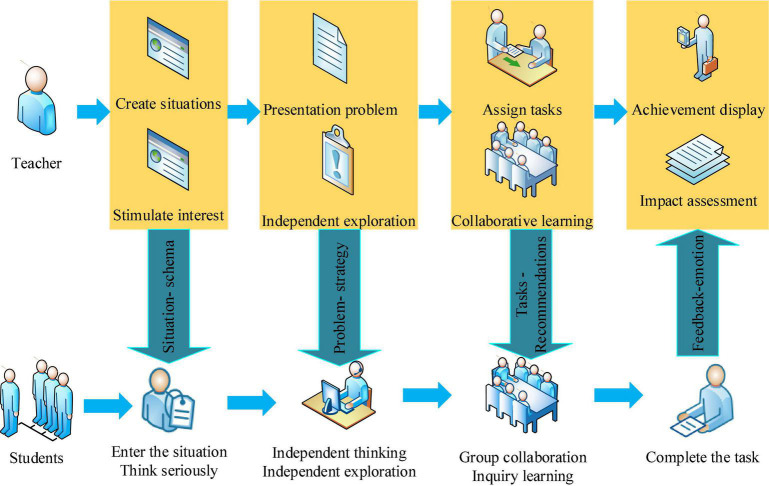
Classroom teaching process.

Teachers summarize and analyze the preview effect of students in the classroom, organize the problems encountered by students, and then design classroom teaching activities according to these problems to create a positive and active learning classroom for students (Narina, 2022). The classroom teaching process is shown in Figure 3.

As shown in Figure 3, creating situations and stimulating interest is that teachers should strive to build situational scaffolding for students in the teaching process, and provide problem scaffolding to guide students to enter the learning situation with problems and tasks, so as to solve the problems encountered in the learning process and develop new learning ability (Chen et al., 2022). Assigning tasks and assisting learning means that teachers build task brackets for students, assign learning tasks to students according to the needs of classroom knowledge content, and students conduct group inquiry learning, creating a relaxed learning atmosphere and relieving their anxiety. After completing the task, the effect displayed is that the teacher builds feedback support for the students, conducts a comprehensive evaluation of each student’s learning effect, helps students gain a sense of accomplishment, and reduces anxiety in the Chinese language and literature major education (Shinta, 2022).

#### Post-class teaching

**FIGURE 4:**
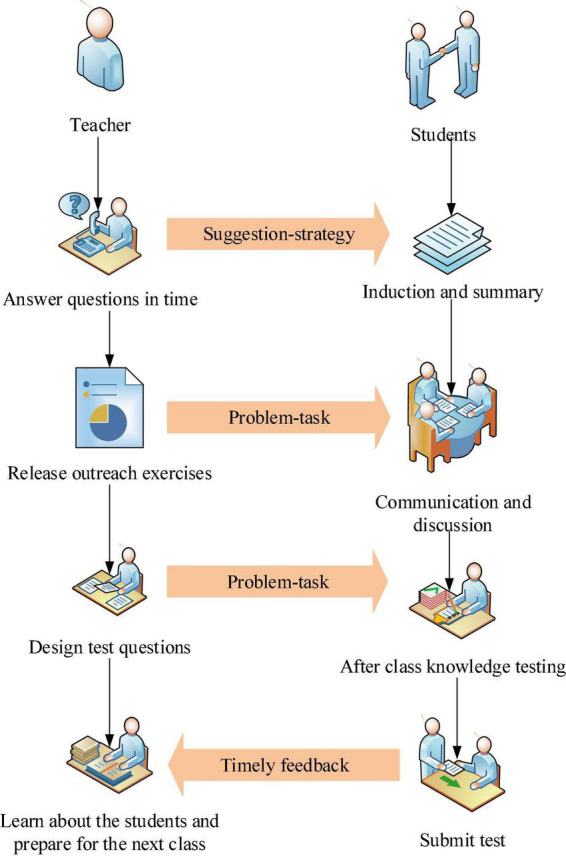
The post-school teaching process.

To fully grasp the knowledge learned in the classroom, it is necessary to constantly review the knowledge. Therefore, teachers should build task scaffolds for students in time, and arrange after-class review tasks for students to help students consolidate what they have learned promptly. The post-school teaching process is shown in Figure 4.

Figure 4 shows that firstly, the induction and summary are the students’ summaries of the knowledge points learned in the classroom. The teacher asks the leaders of each group to publish the excellent notes and course resources of the members of the group to the CCtalk learning platform to realize the sharing of resources. Secondly, research on difficult problems and knowledge expansion mean that teachers organize students to discuss unsolved problems in the classroom in time after class, and arrange expansive learning tasks on the platform to cultivate students’ divergent thinking, thereby completing the transfer of knowledge (Chun and Cennamo, 2022). Finally, in order to test the students’ learning effect on the whole teaching activities, the teacher designs the after-school knowledge test. The students complete the test and submit it on the learning platform. The teacher effectively arranges the teaching design activities of the next class according to the students’ test results and the feedback (Zuhra et al., 2022).

### Experimental investigation data verification

The study used an independent *t*-test to analyze the differences between the two groups of data, and the *t*-test equation is shown in Equation 1:


(1) $$\text{t}=\frac{\bar{{\text{X}}_{1}}-\bar{{\text{X}}_{2}}}{\sqrt{\frac{\sigma_{{\text{X}}_{1}}^{2}+\sigma_{{\text{X}}_{2}}^{2}-2\,\gamma \,\sigma_{{\text{X}}_{1}}\,\sigma_{{\text{X}}_{1}}}{\text{n}-1}}}$$

In Equation 1: $\bar{{\text{X}}_{1}}$, $\bar{{\text{X}}_{2}}$ are the mean of the two samples, respectively; $\sigma_{{\text{X}}_{1}}^{2}$, $\sigma_{{\text{X}}_{2}}^{2}$ are the variances of the two samples; γ is the correlation coefficient of the relevant samples.

At the same time, the variance test is performed on the experimental data, and the test statistic W is shown in Equation 2:


(2) $$\text{W}=\frac{\left(N-k\right)\sum_{\text{i}=1}^{\text{k}}{\left(\bar{{\text{Z}}_{\text{i}}}-\bar{\text{Z}}..\right)}^{2}}{\left(k-1\right)\,\sum_{\text{i}=1}^{\text{k}}\sum_{\text{j}=1}^{\text{N}}{\left(\bar{{\text{Z}}_{\text{ij}}}-\bar{{\text{Z}}_{\text{i}}}\right)}^{2}}$$

In Equation 2: W is the test statistic, k is the number of sample groups, N is the sum of the sample sizes, Z_ij_ is the new variable value after the original data has been transformed. The definition of Z_ij_ can be divided into the following three types:


(3) $${\text{Z}}_{\text{ij}}=\left|{\text{Y}}_{\text{ij}}-\bar{{\text{Y}}_{\text{i}}}\right|$$

In Equation 3: Y_ij_ is the original data; $\bar{{\text{Y}}_{\text{i}}}$ is the arithmetic mean of the i-th sample in the original data.


(4) $${\text{Z}}_{\text{ij}}=\left|{\text{Y}}_{\text{ij}}-\tilde{{\text{Y}}_{\text{i}}}\right|$$

In Equation 4: $\tilde{{\text{Y}}_{\text{i}}}$ is the median of the i-th sample in the original data.


(5) $${\text{Z}}_{\text{ij}}=\left|{\text{Y}}_{\text{ij}}-\tilde{\bar{{\text{Y}}_{\text{i}}}}\right|$$

Cronbach’s Alpha coefficient was used to test the internal consistency of the questionnaire. The equation between the measured value x and the true value is shown in Equation 6:


(6) x=ατ+e

In Equation 6: e is the error value, then the equation for calculating Cronbach’s Alpha coefficient is:


(7) $$\rho_{\tau }=\frac{\text{k}}{k-1}\,\left[1-\frac{\sum_{\text{i}=1}^{\text{k}}\mathrm{var}\,\left({\text{x}}_{\text{i}}\right)}{\mathrm{var}\,\left(X\right)}\right]$$

In Equation 7: var(X) is the variance of the sum of all measurements; var(x_i_) is the variance of each measurement.

## Experimental design and performance evaluation

### Experimental materials

**FIGURE 5:**
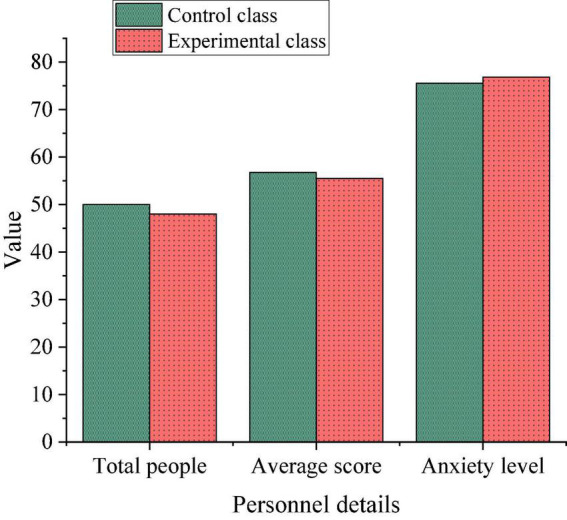
Class basic situation.

The object is two classes of sophomore Chinese language and literature majors at Shaanxi Normal University in Xi’an, one of which is the control class and the other is the experimental class. These two classes belong to parallel classes, and the teaching materials and teachers are the same. The basic situation of the two classes is shown in Figure 5.

As shown in Figure 5, the total number of the control class is 50, and the total number of the experimental class is 48; The average score of the control class is 56.74, and that of the experimental class is 55.48; The anxiety of the control class is 75.53, and the anxiety of the experimental class is 76.83.

Meanwhile, drawing on the previous research and the scaffolding reading teaching method designed the Chinese language reading anxiety questionnaire is formulated, and different items are designed according to the scaffolding teaching stage. The Chinese language and literature reading anxiety scale are divided into three dimensions: text comprehension anxiety, emotional anxiety, and cultural background anxiety, with a total of 20 multiple-choice questions. Using the Likert five-point scoring method, assign 1–5 points from “strongly disagree” to “strongly agree.” The total score ranges from 20 to 100, with 20–46 being a low reading anxiety level, 47–68 being a moderate reading anxiety level, and 69–100 being a high reading anxiety level. The higher the student’s score, the higher the anxiety the student has during the reading process.

### Experimental environment

Before the beginning of the experiment, a pre-reading test was conducted on the students of the two classes, and the Chinese language and literature reading anxiety scale was issued to ensure that the reading level and reading anxiety levels of the two classes are within the acceptable range of differences. SPSS24.0 software was used to analyze the test scores and reading anxiety values. During the teaching experiment, the conventional teaching model was adopted in the control class, and the scaffolding instruction mode based on the Internet was adopted in the experimental class. At the end of the experiment, the post-reading test paper and the Chinese Language and Literature Reading Anxiety Scale were distributed again to the two classes. SPSS24.0 software was used to analyze and discuss the data before and after the test.

### Parameters setting

In order to ensure that the Chinese language and literature reading anxiety of the students in the two classes is consistent, descriptive statistics and independent sample *t*-tests are performed on the reading anxiety values of the control class and the experimental class, and the percentage of the confidence interval is set to 95%.

The Cronbach’s Alpha value of the test paper before the experiment is 0.847, and after the experiment is 0.839. The two papers have good internal consistency.

### Performance evaluation

#### Analysis of the results of the reading anxiety scale before the experiment

**FIGURE 6:**
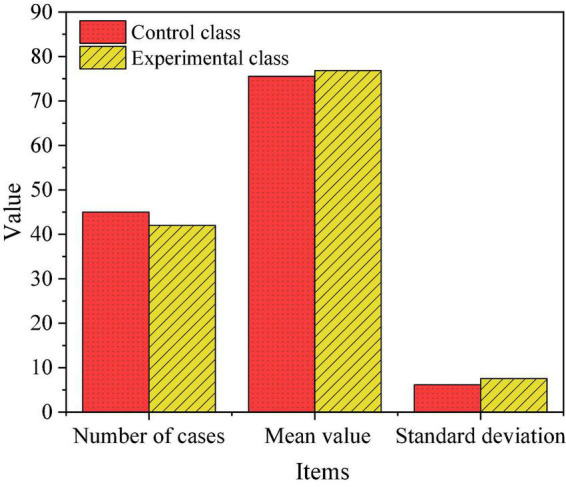
Descriptive statistical results.

Descriptive statistics and independent *t*-tests were performed on the pre-experiment the reading anxiety values of the control class and the experimental class. The results are shown in Figure 6 and Table 1.

**TABLE 1:** Independent test results.

	Levene test		*T*-test		
	*F*	Sig	*t*	*P* (double-stern)	Mean difference
Assume equal variances	0.476	0.482	−0.889	0.368	−1.29726
No equal variance assumed			−0.883	0.371	−1.29726

As shown in Figure 6, before the start of the teaching experiment, the average reading anxiety of the control class was 75.54, and the average anxiety of the experimental class was about 76.83. Although there are some differences in the average anxiety of students in these two classes, the difference is relatively small and almost close. It can be seen from Table 1 that the value of Sig is 0.482 > 0.05, which means that the reading anxiety values of the two classes are homogenous. Therefore, there was no significant difference in the reading anxiety of Chinese language and literature between the two classes before the experiment.

#### Analysis of the results of the reading anxiety scale after the experiment

**FIGURE 7:**
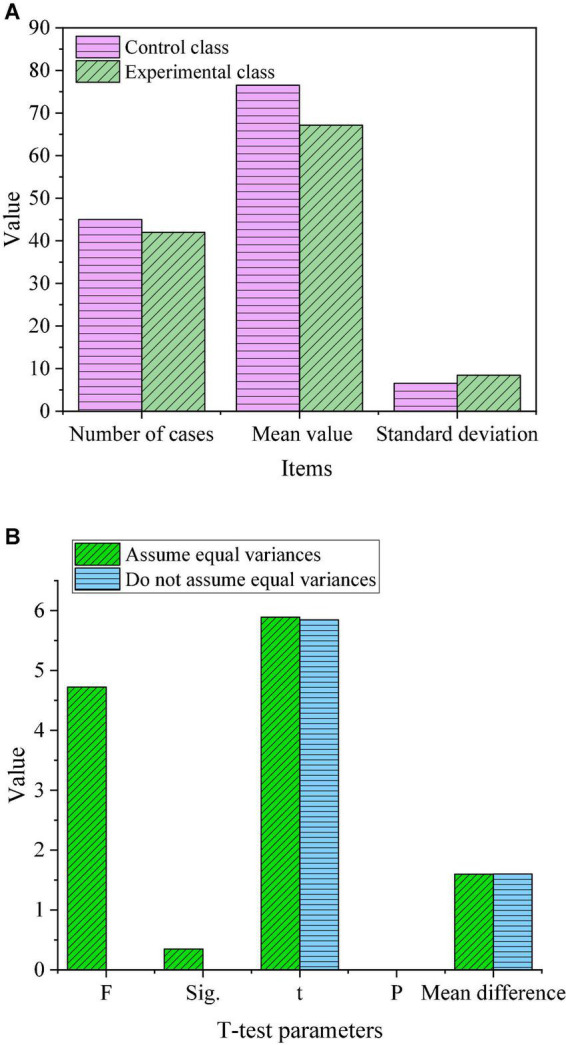
Experimental result analysis. **(A)** Descriptive result. **(B)** Independent *t*-test result.

Descriptive statistics and independent samples *t*-test were performed on the post-test reading anxiety values of the control class and the experimental class (confidence interval percentages were both set to 95%), and the output results are shown in Figure 7.

As shown in Figure 7, after the teaching experiment, the average reading anxiety of the experimental class was 67.13 points, and that of the control class was about 76.52 points. The average post-test reading anxiety of the experimental class was 9.39 points lower than that of the control class. It shows that after the teaching experiment, the Chinese language and literature reading anxiety of the students in the experimental class has been relieved to a certain extent, while the students in the control class may have slightly higher reading anxiety than the pre-test due to the final exam. At the same time, the value of Sig is 0.035 < 0.05, which means that the variance of reading anxiety in the two classes is uneven. From *t* = 5.843, *P* = 0.000 < 0.05, it can be seen that there were significant differences in reading anxiety of Chinese language and literature between the two classes before the experiment.

#### Analysis of test results

**FIGURE 8:**
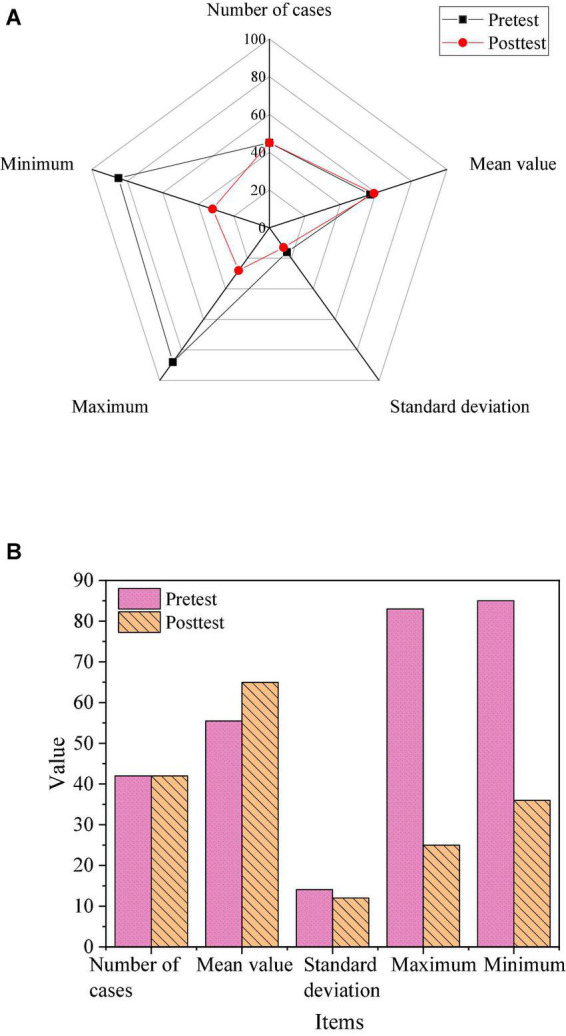
Results of descriptive analysis of grades. **(A)** The results of the control class. **(B)** The results of the experimental class.

**FIGURE 9:**
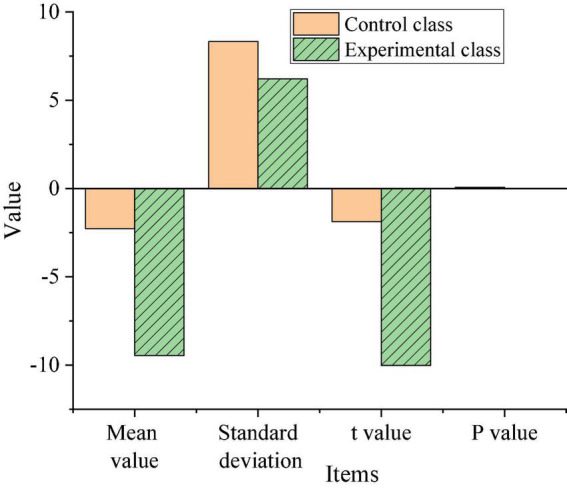
Independent *t*-test result.

The reading test was conducted on the classes after the scaffolding instruction experiment, and the obtained results were subjected to descriptive analysis and independent *t*-test. The specific results are shown in Figures 8, 9.

As shown in Figure 8, the average score of the students in the control class was 56.76 in the pre-test and 58.95 in the post-test; while the average score of the students in the experimental class was 55.48 in the pre-test and 64.95 in the post-test. Students’ reading scores improved by nearly 9 points after the experiment. Although the reading scores of the students in the control class also improved, there was no significant improvement in the reading scores of the students in the experimental class.

As shown in Figure 9, the paired sample *t*-test of the pre-test and post-test of the students in the control class showed *P* = 0.067 (>0.05), indicating that there was no significant difference in the pre-test and post-test scores of the control class. The paired sample *t*-test of the pre-test and post-test of the students in the experimental class showed that *P* = 0.000 (less than 0.05), which shows that the students in the experimental class have significant differences in the pre-test and post-test scores. Therefore, the teaching experiment of the scaffolding instruction model based on educational psychology in the experimental class was successful, which improved the students’ reading performance.

## Discussion

The rapid development of computer and network technology has injected new impetus into education. Integrating the teaching of the Chinese language and literature into the network platform is not only the demand of the era of educational informatization but also an inevitable choice in line with the development of the times. Reading ability is one of the important criteria to measure the education of Chinese language and literature majors for college students. However, most students are generally anxious when reading Chinese language and literature articles, which leads to poor reading results and reduced learning efficiency for Chinese language and literature majors. In order to alleviate or eliminate students’ anxiety about reading Chinese language and literature to a greater extent, the scaffolding teaching model is applied based on educational psychology to college Chinese language reading teaching for experimental research, and the validity of the model is confirmed through case analysis. The research results show that college students generally have Chinese language reading anxiety in the process of learning and reading. The teaching effect of the scaffolding teaching mode based on the internet and educational psychology is significant. Using the new teaching mode to guide college students’ Chinese language reading class is helpful to improve students’ Chinese language reading performance. In addition, the students in the experimental class have enhanced classroom participation and can actively complete the learning tasks assigned by the teacher. Therefore, in the subsequent teaching process, scaffolding teaching should be combined with Chinese language reading teaching as much as possible to improve students’ classroom participation and reading enthusiasm to help students better accept Chinese language knowledge. Compared with previous research results, the research results focus on the analysis of the Chinese language and literature reading. Meanwhile, from the perspective of teaching psychology, it can help to better analyze students’ psychology, so the research results are more innovative and representative.

## Conclusion

### Research contribution

From the perspective of educational psychology, through the combination of educational psychology and Internet technology, a kind of scaffolding Chinese language and literature professional education method was put forward based on educational psychology. At the same time, the students of two classes in a university in XX city were selected as the research objects, and a one-term teaching experiment was carried out. The data of reading results of Chinese language and literature in two classes were collected through questionnaires and tests, and the data were analyzed by SPSS24.0 software after the experiment. The results show that after the teaching experiment, the average reading anxiety of Chinese language and literature in the experimental class is 9.39 points lower than that in the control class, and the reading anxiety of Chinese language and literature in the experimental class is alleviated to some extent. The Chinese language and literature test scores of the students before and after the teaching experiment were compared using the scaffolding instruction model, and the paired sample *t*-test was performed on the scores. The results showed that the significant indigenous value of the paired *t*-test of the students before and after the teaching experiment was *P* = 0 (<0.05), indicating that the pre-test and post-test scores of the students in the experimental class were significantly different. It can be seen that students’ Chinese language and literature scores improved after the experiment. Therefore, the scaffolding instruction mode based on educational psychology can be used in the teaching of Chinese language and literature. This study can provide relevant innovative suggestions for the teaching of Chinese language and literature, and provide a reference for subsequent research.

### Future works and research limitations

The deficiency of this study is that the research cycle is short, the experimental study only lasted for one semester, and the improvement in reading performance is not large. In further research, the research content and scope can be expanded, the experimental cycle can be extended, and the credibility of the practical effect of the scaffolding instruction mode based on the Internet and educational psychology can be improved.

## Data availability statement

The raw data supporting the conclusions of this article will be made available by the authors, without undue reservation.

## Ethics statement

The studies involving human participants were reviewed and approved by Heilongjiang Bayi Agricultural University Ethics Committee. The patients/participants provided their written informed consent to participate in this study. Written informed consent was obtained from the individual(s) for the publication of any potentially identifiable images or data included in this article.

## Author contributions

The author confirms being the sole contributor of this work and has approved it for publication.
